# *In situ* Parallel Training of Analog Neural Network Using Electrochemical Random-Access Memory

**DOI:** 10.3389/fnins.2021.636127

**Published:** 2021-04-08

**Authors:** Yiyang Li, T. Patrick Xiao, Christopher H. Bennett, Erik Isele, Armantas Melianas, Hanbo Tao, Matthew J. Marinella, Alberto Salleo, Elliot J. Fuller, A. Alec Talin

**Affiliations:** ^1^Sandia National Laboratories, Livermore, CA, United States; ^2^Sandia National Laboratories, Albuquerque, NM, United States; ^3^Department of Materials Science and Engineering, Stanford University, Stanford, CA, United States

**Keywords:** analog memory, organic electrochemical transistor, in-memory computing, ECRAM, on-line training, outer product update

## Abstract

In-memory computing based on non-volatile resistive memory can significantly improve the energy efficiency of artificial neural networks. However, accurate *in situ* training has been challenging due to the nonlinear and stochastic switching of the resistive memory elements. One promising analog memory is the electrochemical random-access memory (ECRAM), also known as the redox transistor. Its low write currents and linear switching properties across hundreds of analog states enable accurate and massively parallel updates of a full crossbar array, which yield rapid and energy-efficient training. While simulations predict that ECRAM based neural networks achieve high training accuracy at significantly higher energy efficiency than digital implementations, these predictions have not been experimentally achieved. In this work, we train a 3 × 3 array of ECRAM devices that learns to discriminate several elementary logic gates (AND, OR, NAND). We record the evolution of the network’s synaptic weights during parallel *in situ* (on-line) training, with outer product updates. Due to linear and reproducible device switching characteristics, our crossbar simulations not only accurately simulate the epochs to convergence, but also quantitatively capture the evolution of weights in individual devices. The implementation of the first *in situ* parallel training together with strong agreement with simulation results provides a significant advance toward developing ECRAM into larger crossbar arrays for artificial neural network accelerators, which could enable orders of magnitude improvements in energy efficiency of deep neural networks.

## Introduction

Machine learning and artificial neural networks have promising applications in diverse fields (Lecun et al., 2015). Such algorithms are very energy intensive to implement in conventional digital computers. The energy intensity arises from the need to shuttle large quantities of information from memory to processor to conduct large matrix multiplications and to update matrix weights (Marinella et al., 2017; Sze et al., 2017). Crossbar arrays of analog non-volatile memory elements eliminate this “memory wall” and promise to reduce the energy consumption of inference and training by conducting matrix computations locally at the memory elements (Nawrocki et al., 2016; Burr et al., 2017; Ielmini and Wong, 2018; Xia and Yang, 2019). Hardware inference accelerators for analog matrix vector multiplication, also known as dot-product engines, have been demonstrated using non-volatile memory like inorganic memristors (Yao et al., 2017; Hu et al., 2018; Li et al., 2018b; Yao P. et al., 2020; Yeon et al., 2020), organic memristors (Emelyanov et al., 2016; Lin et al., 2016; Park and Lee, 2017), phase-change memory (Ambrogio et al., 2018; Nandakumar et al., 2020; Sebastian et al., 2020), and floating gate memory (Guo et al., 2017; Lee and Lee, 2020), and may be commercialized in the coming years.

While there exist several potential hardware solutions for analog inference, *in situ* training accelerators based on analog memory have been extremely challenging to implement. Most analog non-volatile memory devices suffer from nonlinear and unpredictable conductance updates as well as high device-to-device variation, which reduces training accuracy (Agarwal et al., 2016). Training accelerators should also have the ability to update all weights in parallel through outer product updates to minimize latency and energy consumption (Agarwal et al., 2016; Gokmen and Vlasov, 2016). While this is achievable in very small crossbar arrays (Alibart et al., 2013), high write currents in the μA or mA preclude this ability in larger arrays due to voltage drops across the write lines. Instead, *in situ* training utilize sequential device-by-device weight updates (Prezioso et al., 2015; Bayat et al., 2018; Li et al., 2018a; Cai et al., 2019), resulting in extra latency and energy consumption for larger array sizes between 10^3^ and 10^6^ devices.

Three-terminal electrochemical random-access memory (ECRAM), also known as redox transistors, can address the accuracy, energy, and latency deficiencies of two-terminal memristors (Fuller et al., 2017, 2019b; van de Burgt et al., 2017; Sharbati et al., 2018; Tang et al., 2018; Kim et al., 2019; Li et al., 2019, 2020a,b; Melianas et al., 2020; Tuchman et al., 2020; Yao X. et al., 2020). ECRAM achieves exceptionally reproducible, linear, and symmetric weight updates by encoding information in resistance values that reflect changes in the average bulk concentration of dopants like protons in transistor-like channels. ECRAM can also achieve massively parallel weight updates: because most ECRAMs operate at room temperature and do not need joule heating for switching. The switching energy and switching current is expected to be below 1 fJ and 1 nA, respectively, for scaled devices (Fuller et al., 2017; van de Burgt et al., 2017; Sharbati et al., 2018; Li et al., 2020a). As a result, voltage drops along write lines are negligible, so all devices in the crossbar sense the same write voltage even when many devices are updated simultaneously. This enables massively parallel weight updates even when scaled to larger crossbar arrays. The ability to update all weights in parallel via an outer product is crucial for realizing analog training accelerators that substantially exceed the performance of digital ones (Fuller et al., 2019a).

While parallel weight updates have been shown using ECRAM (Fuller et al., 2019a; Kim et al., 2019), *in situ* training utilizing this outer product update has not been demonstrated due to overlapping fabrication and systems engineering challenges. Instead, numerical simulations (Agarwal et al., 2016; Jacobs-Gedrim et al., 2017; Bennett et al., 2019a; Kim et al., 2019) based on the switching properties of just one or few devices have been used to predict the accuracy of training large crossbar arrays (Fuller et al., 2017, 2019a; van de Burgt et al., 2017; Li et al., 2019, 2020a). Without experimental validation, it is unclear if such numerical simulations will accurately capture experimental training protocols, especially in the presence of non-ideal device behavior, variations between devices, loss of state, or sneak current pathways that are difficult to account for in array simulations using individual device measurements.

In this work, we experimentally train a small crossbar array of ECRAM cells in parallel with high efficiency alongside accuracies close to software-derived values at floating point precision. This is not only an advancement for ECRAM, but also provides the first experimental realization of scalable and parallel *in situ* (on-line) training utilizing outer product updates of stochastic gradient descent conducted through in-memory computing. Moreover, we also show near-perfect agreement between crossbar array simulations and experimental training results: we not only replicate the number of epochs to convergence, but also the exact evolution of conductance weight updates. This result validates these training models and provide strong evidence that ECRAM cells can accurately execute deep neural network algorithms as predicted from crossbar simulations. The highly accurate training and excellent agreement with simulation result from the linear and deterministic switching of ECRAM devices. We also consider the importance of these characteristics for both spiking and deep neural networks. By both demonstrating parallel *in situ* training and validating numerical simulations, we show the potential of using three-terminal ECRAMs as a platform to design in-memory hardware accelerators for efficient *in situ* training of artificial neural networks.

## Results and Discussion

### Device Fabrication and Circuit Design

**FIGURE 1:**
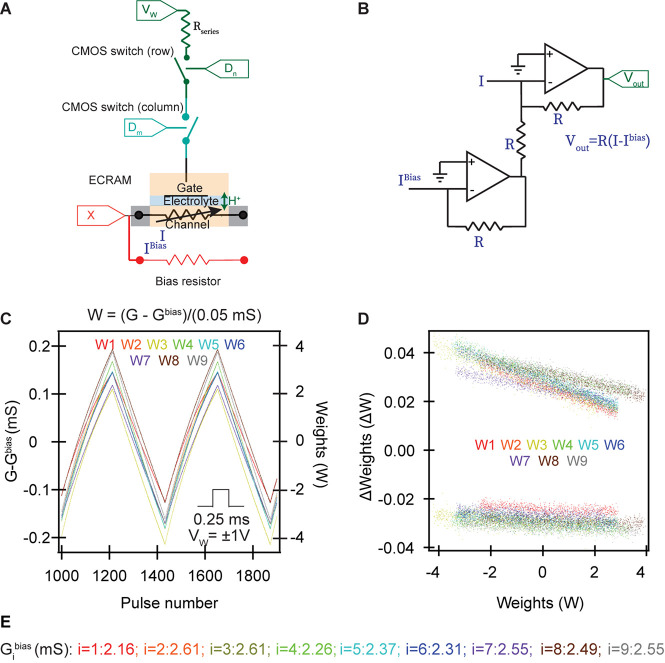
Organic ECRAM cells as electrochemical synapses. **(A)** Each synapse in the crossbar array contains one ECRAM, a discrete bias resistor used to obtain negative weights, a series resistor to control the gate current, and two CMOS switches as selectors. The ECRAM cells store synaptic weights by electrochemical doping/de-doping of the channel, which alters its electronic conductivity. When both switches are ON, the write current programs the redox transistor to the desired state. When either or both switches are OFF, the ECRAM cells retain their state. Two switches are used to accommodate the outer product update. **(B)** Subtractor circuit used to calculate the difference in the conductance between the ECRAM channel and the bias resistor; the same circuit is used when the currents from multiple devices are summed in an array. 10 kΩ resistors were used for R_series_ in **(A)** and all resistors in **(B)**. **(C)** Potentiation and depression of nine synapses, using pulses of identical magnitude. The resulting current (positive or negative) is used to calculate $\left(G_{i}-G_{i}^{b\,i\,a\,s}\right)$, where *G_i* is the electronic conductance of ECRAM *i* and $G_{i}^{b\,i\,a\,s}$ is the conductance of the bias resistor, as written in **(E)**. The non-dimensional weight is computed during post-processing by $W_{i}=\left(G_{i}-G_{i}^{b\,i\,a\,s}\right)$/(0.05*mS*). **(D)** Device-to-device variation of the nine synapses, showing the weight update for different starting weight values. Each device completed 50 full cycles, where each cycle equals 440 weight updates, equally divided amongst potentiation and depression. For clarity, a random 10% subset of the ∼20,000 weight updates for each device are shown. **(E)** The conductance values of the fixed discrete bias resistors.

We fabricate nine organic ECRAM cells using PEDOT:PSS as the mixed ionic-electronic conduction weight storage element and PVDF-HFP combined with EIM:TFSI as the ion gel electrolyte (Melianas et al., 2020). Weight updates are conducted when electrons and charge-compensating ions are moved between the gate and channel electrodes; based on past work on organic ECRAM (van de Burgt et al., 2017; Fuller et al., 2019a), we anticipate that the dominant charge-compensating ion is the proton, although it has not yet been proven for this device (Melianas et al., 2020). The weights are read using the electronic conductance of the channel. An electrochemical synapse contains an ECRAM cell paired with a fixed bias resistor, a series resistor, and two CMOS switches (MAX327CPE) as selectors (Figure 1A). The conductance of the ECRAM cell stores the synaptic weights. Because (deep) neural network uses both positive and negative weights, while electronic conductances can only be positive, we enable negative weights by subtracting the current between the ECRAM and the bias resistor (Figure 1B; Agarwal et al., 2016). Other non-volatile memory cells typically use the difference between two memory elements to store a single synaptic weight (Burr et al., 2017). We use a discrete through-hole resistor (Yageo) with a fixed resistance like 392 or 402 Ohms as bias resistors; the conductance values of the fixed bias resistors (G^bias^) parallel to the ECRAM channel are written in Figure 1E.

Because large, rapid changes in the ion concentration in conducting polymers could result in rapid swelling and mechanical delamination, we utilize a series resistor to control the amount of current flowing into the ECRAM’s gate (Keene et al., 2018a; Fuller et al., 2019a). This series resistor is not needed in inorganic ECRAM cells because higher ionic resistance of the inorganic solid electrolyte is sufficient to limit the current (Fuller et al., 2017; Li et al., 2020a). The two CMOS switches are used to select the device to be programmed: weight updates are conducted only if both CMOS switches are ON; as we show later, one of these switches selects the rows and the other selects the columns that undergo weight updates, enabling parallel outer product updates of all devices in just one or two steps. This selection scheme differs from our past work utilizing a two-terminal diffusive memristor selector (Fuller et al., 2019a). While two CMOS switches will likely require larger chip area, it also results in more accurate and reliable switching by not using the more stochastic diffusive memristor. It also eliminates the extra “read-selector” CMOS switch previously used (Fuller et al., 2019a). In terms of functionality, the CMOS switches can be replaced with a single transistor switch, so the final synapse design is two transistor switches, one ECRAM, an offset bias resistor (or memristor) and one series resistor.

The sign of V_W_, common to all devices, controls the direction of the weight update, either potentiation (increase conductance) or depression (decrease conductance). A subtractor circuit is used to subtract the current between the channel and the bias resistor based on a proposed architecture in ref. (Agarwal et al., 2016; Figure 1B). The resulting $I_{i}-I_{i}^{b\,i\,a\,s}$ current is measured using an operational transconductance amplifier, from which we can calculate $G_{i}-G_{i}^{b\,i\,a\,s}$ by dividing this current from the input V_R_.

The linearity and reproducibility of the weight updates for the nine organic ECRAM cells are given in Figures 1C,D. The $G_{i}-G_{i}^{b\,i\,a\,s}$values are plotted in Figure 1C, where *i* indicates the index of the ECRAM cell. Using 0.25 ms write pulses, we obtain over 200 analog states for each device within the conductance range – the number of analog states in these devices can be changed by modifying the write time (Melianas et al., 2020). The equilibrium conductance *G_i* while the gate and channel are shorted is about 2.5 mS with some device-to-device variation. The conductance values of the bias resistors are listed within Figure 1. Although more desirable low-conductance devices in the nano-Siemens range were previously realized (Fuller et al., 2019a), this study used the higher conductance devices with greater fabrication reproducibility for proof-of-concept demonstration.

To obtain the non-dimensional synaptic weight *W_i*, we divide $G_{i}-G_{i}^{b\,i\,a\,s}$ by 0.05 mS, and plot the values on the right axis of Figure 1C. In Figure 1D, we show the change in weight upon each applied write pulse, demonstrating low cycle-to-cycle and device-to-device variation across the nine ECRAM devices. An interesting feature is the higher slope, or nonlinearity, of ΔW vs. W upon potentiation over depression. One contributing factor is the ECRAM cells in the low-conductance state lose state faster due to oxidation with the ambient environment than cells in high-conductance states (Keene et al., 2018b; Melianas et al., 2020). Other electrochemical and electronic mechanisms also contribute to this nonlinearity and are subjects for future investigations.

**FIGURE 2:**
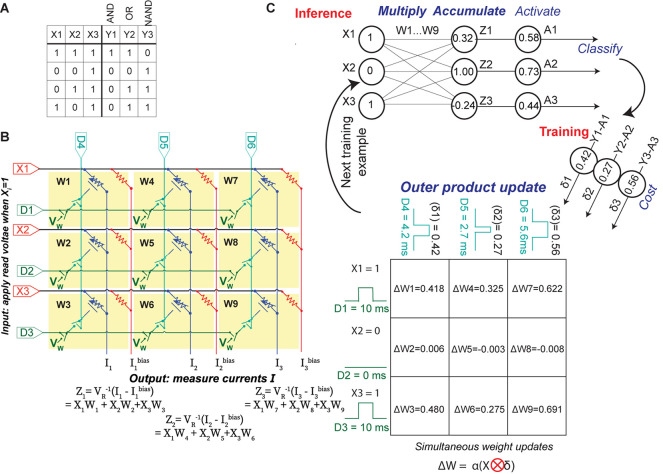
Implementation of parallel array learning. **(A)** List of the training examples in this work. The “X” represents the inputs fed into the three rows of the crossbar, while the outputs “Y” show the correct solutions and used to compute the cost functions of each column output. **(B)** Circuit schematic of the nine-ECRAM array. Each synapse W_1__–__9_ contains an element in Figure 1A; the series resistor is not shown here for legibility. During inference, the circuit measures I-I^*bias*^ using the subtractor circuit in Figure 1B, and this is converted to the non-dimensional software value Z. During weight updates, the digital inputs D_1__–__6_ are used to select the time that the switches are ON in each row and column, which controls the change in weight for each device in accordance with an outer product update. **(C)** Flow diagram of learning process. During inference, multiply and accumulate are conducted using the crossbar in the manner described in **(B)**. Afterward, the activation energy and cost functions are computed in software and used to compute the weight update. To conduct a parallel outer product update, the pulse widths of digital switches D_1__–__3_ correspond to the inputs X_1__–__3_, while the pulse widths of digital switches D_4__–__6_ correspond to the cost δ_1__–__3_. Positive and negative weight changes must be conducted separately, so two steps are needed to conduct a parallel weight update.

We conduct parallel in-situ training of a simple perceptron network with two binary inputs (X1 and X2) and a bias input X3, which is always equal to 1. We use three outputs corresponding to the three logic gates that are trained in this experiment (Figure 2A); these represent three of the four linearly separable elementary logic gates containing two binary inputs, one bias input, and one output. For each logic gate, the training set consists of four examples corresponding to all possible values of X1 and X2; the training set is consecutively and iteratively used to train the ECRAM array until the network correctly identifies the entire training set for all logic gates. These logic gates were chosen due to the low number of devices available, which is a common challenge for emerging device technologies where fabrication processes have not been fully established.

Figures 2B,C shows the circuit and the flow diagram for both forward and backward propagation step based on stochastic gradient descent. The nine electrochemical synapses are electrically arranged into a 3 × 3 crossbar using a printed circuit board. To conduct forward propagation, or inference, the crossbar uses multiply-and-accumulate operations to perform the matrix operation ***Z*** = *W*^*T*^**X**, where W is the matrix of synaptic weights and **X** is the vector of input value. We apply a small read voltage (V_R_) ∼50 mV to represent a logical “high” at the input and measure the accumulated current at the bottom of each column, which is held at virtual ground using an operational amplifier. The vector-matrix product **Z** is proportional to the difference between the accumulated current of the ECRAM column and the accumulated current through the bias resistor column. It is computed by using a subtractor circuit (Figure 1B) in each column. Once this vector-matrix product is obtained, we compute the sigmoid activation function (***A* = σ (Z)**) in software and compare the result to 0.5 to obtain the binary prediction. The error δ equals the solution **Y** minus the activation **A:** if the absolute value of δ for a column is less than 0.5, the network has correctly classified that column; if |δ| > 0.5, then the network has incorrectly classified the column.

Next, we describe how to train the network in parallel. The change in weight for the array is given by the outer product update $\Delta \,W=\alpha \,\left(\text{X}\,⊗\frac{\partial ℒ}{\partial Z}\right)$ where ⊗ denotes the outer product, $\frac{\partial ℒ}{\partial Z}$ is the derivative of the cost function ℒ=**Y***log*⁡(**A**) + (1−**Y**)log(1−**A**) with respect to the vector-matrix product **Z**, and α is the learning rate. We choose cross-entropy as the cost function such that $\frac{\partial ℒ}{\partial Z}$ **= Y-A =** δ. In addition to the continuous-valued weight update, we also incorporated a discrete-value cross-entropy update scheme whereby we rounded δ to take the value of −1, 0, or 1 during weight updates. We demonstrate these two weight update schemes to show the broader generalizability of our approach.

To conduct the parallel outer product update, we simultaneously apply three sets of voltages to the circuit (Figure 2B). First, we apply a write voltage *V*_*W*_ = 1V or −1V to all the synapses. Second, we apply a pulse to the “row” digital terminals D_1__–__3_ that equals 10 ms when *X*_*i*_ = 1 and 0 ms when *X*_*i*_ = 0; we note that these pulse widths can also be analog rather than binary. Third, we apply a variable-width pulse to the “column” digital terminals D_4__–__6_ that is proportional to α⋅δ for continuous-valued updates and α⋅round(δ) for discrete-value updates; α⋅δ=1 correspond to 10 ms. Since weight updates entail that both switches are ON, the weight of a synapse does not change when *X*_*i*_ = 0 during that training step. In other words, this pulse timing conducts an outer product multiplication between the input **X** on the “row” terminals and α⋅δ or α⋅round(δ) on the “column” terminals (Figure 2C).

This parallel weight update scheme is scalable to larger arrays: the outer product update enables all weights in an array to be updated by controlling the D terminals at the edges of the array, regardless of array sizes. Because the V_W_ terminal is shared among all devices, two sequential sets of pulses are needed to update all weights, one to increase conductance weights (*V*_*W*_ = −1V) and one to decrease weights (*V*_*W*_ = 1V); we note that, if the inputs X_i_ can be negative, then four update cycles would be needed. The CMOS switches are used for selectivity and to prevent crosstalk: when the switches are OFF, the gates are electrically isolated from each other. The devices are also ionically isolated from each other during fabrication. In our training experiments, all values of *W* were collected for post-mortem processing and analysis, but this information was not used during training in order to demonstrate its parallelism.

The table in Figure 2C shows one example step of this parallel weight update using the continuous-valued cross-entropy cost function. This scheme enables accurate analog weight updates for all devices in parallel, where the changes in the device’s conductance nearly perfectly match the desired values; this will be quantified in detail later.

### Experimental *in situ* Parallel Training

**FIGURE 3:**
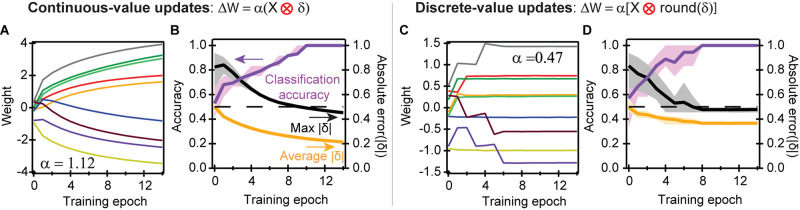
Convergence of perceptron operations using different learning rules. **(A)** Weight updates of the nine synapses using stochastic gradient descent to solve for AND, OR, and NAND logic gate functions. The cross-entropy cost function with continuous-value weight updates was used, such that $\frac{\partial ℒ}{\partial Z}$ = Y-A = δ. **(B)** Average accuracy and absolute error to train six different initial seed values; the shaded areas represent the range of values taken by the six seeds. It takes about ten training epochs to fully classify the problem. Our learning rule states that an absolute error |δ| less than 0.5 is a correct classification. The average absolute error takes the mean absolute error |δ| of the 12 operations defined in Figure 2A in each training epoch; the max error is the maximum error of the 12 classification operations. Convergence is defined as when the classification accuracy is 100%, or when the maximum error is less than 0.5. **(C)** Experimental training when using discrete-value weight updates: positive, negative, or 0. **(D)** Convergence, accuracy, and error for discrete-value updates, with the same colors defined in **(B)**. The colors in **(A,C)** are the same as the ones defined in Figure 1C.

The four training examples, each using three logic operations (Figure 2A), are consecutively and iteratively fed into the network, until the network attains 100% accuracy as determined by when absolute errors |δ| decrease below 0.5 for all cases in the training set. Figure 3 shows the results of the training. After randomly seeding values for the initial condition, we show the evolution of synaptic weights in Figure 3A, where we utilize the continuous-value weight updates given by the cross-entropy cost function, after each training epoch. The updates to the weights decrease over time, signifying convergence. While the threshold for accuracy occurs when |δ| < 0.5, continued training epochs further decrease the error, which provide a margin against noise and other non-idealities.

Figure 3B plots the fraction of twelve operations (four training examples multiplied by the three logic gates in Figure 2A) that were accurately classified, the average absolute value of the error (|δ|) across all twelve operations, and the maximum |δ| across the twelve operations. Figure 3B incorporates the results of six different random initial seeds: the dark lines equal the average values for all six seeds, while the lighter shaded areas equal the full range for the different seeds. When the maximum |δ| is less than 0.5, then all input cases representing the logical function have been correctly learned by the corresponding ECRAM devices. Our results show a gradual reduction in error, and an average of eight training epochs to reach the full classification accuracy.

Discrete-value weight updates result in somewhat different convergence behavior. Rather than slowly converging like the continuous case, the weights stop updating once all computed values of |δ| are below 0.5, and the problem is correctly classified (Figure 3C); changes in weight after this point reflect non-idealities, such as slight drift or rebound after weight weigh updates. This rebound may occasionally cause a temporary drop in classification accuracy when the weights are near the threshold of accuracy; this is quickly corrected during the next weight update.

While discrete-value weight updates achieve rapid convergence, the convergence rate is also more sensitive to the initial conditions. This results in a higher variability of the error and accuracy, as shown by the larger spread of the lighter shaded regions in Figure 3D. Because discrete-value weight updates have sharp ending thresholds, certain operations reside at the threshold of accuracy where δ_i_∼0.5, providing less margin against possible drift, noise, and memory loss than the continuous-valued updates (Figure 3A).

One important difference is that the discrete-valued updates converge somewhat faster than continuous-valued updates. The reason is because discrete-value updates only update weights during incorrect classification, whereas continuous-value updates also update weights during correct classification, which may result in updates in the “wrong” direction.

As an example, suppose we solve for the OR gate when *W4* = *W5* = *W6* = 0.4; a value of *Z* > 0 ultimately corresponds to logical 1 and *Z* < 0 to logical 0. This correctly classifies the first three training examples in Figure 2A but incorrectly classifies the fourth (*X1* = *X2* = 0). During discrete-value updates, only the incorrectly classified fourth training example will trigger weight updates. Full convergence is reached as soon as *W6* < 0, which can happen in just a single training epoch. In contrast, during continuous-value updates, W4, W5, and W6 will increase during the first three training examples because 0 < |δ| < 0.5. These increases in W6 will counterbalance the decrease during the fourth training example, resulting in slower convergence. This process also increases the magnitude of W4 and W5, explaining why the weights are generally larger in the continuous-valued rather than discrete-valued updates. Another reason for the higher weight ranges of Figure 3A is because the weights continue to change even after convergence. Despite these differences, our training scheme can correctly classify the logic gates to full accuracy after several training epochs, for all six seed values initiated.

### Crossbar Simulations Quantitatively Reproduce Experimental Data

Next, we conduct crossbar simulations using the same initial conditions to compare against experimental results. Two types of simulations are conducted. The “simulated” result accounts for nonlinearity, cycle-to-cycle variation, and device-to-device variation within the array by interpolating the switching data in Figures 1C,D to predict the results of each weight update for all nine devices. The “ideal numerical” result presents the values at the floating point precision of a simulated neural network.

**FIGURE 4:**
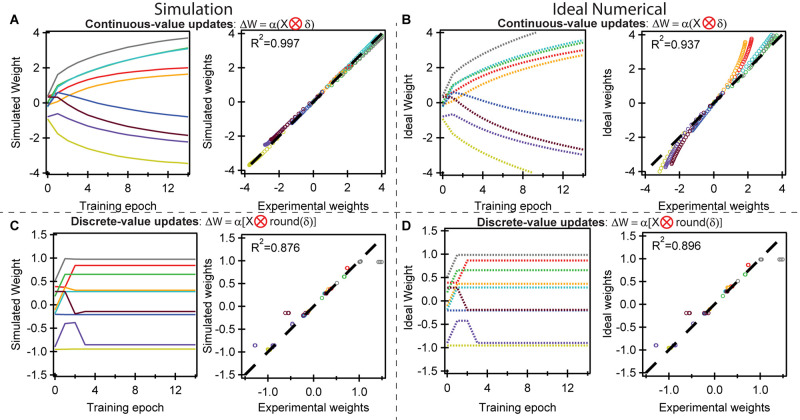
Simulated training behavior compared to the experimental behavior. **(A)** (left) Simulated weight evolution using the switching properties measured in Figures 1C,D. The results are essentially identical to the experimental results in Figure 3A. (right) A plot of the experimental vs. simulated weights show an *R*^2^ = 0.997, demonstrating that the simulations can fully replicate in-situ experimental training procedures. The weights across all epochs are plotted. **(B)** Ideal numerical weight evolution for the logic gates assuming perfectly linear and symmetric devices at floating point precision. There exist some deviations at higher and lower weights as a result of nonlinear and asymmetric switching behavior at the extremes of dynamic range in experimental ECRAM devices. **(C,D)** Comparison of simulated, ideal, and experimental results using the discrete-value rather than continuous-value weight updates. These show more deviations than **(A,B)** because slight variations in weights may result in an extra, relatively large weight update in the experimental results. All colors have the same meaning as in Figure 1C.

Figure 4A shows the simulated weight evolution compared with the experiment using continuous-value weight updates based on the cross-entropy cost function. The agreement is near perfect, with a coefficient of determination *R*^2^ = 0.997. This agreement results from the low cycle-to-cycle variability and the highly deterministic and predictable switching behavior, such that the switching behavior during training is essentially identical to the switching behavior during ramping in Figures 1C,D. This enables quantitative agreement between the experimental and simulated conductance weight on each device. Figure 4B compares the experimental results to the ideal numerical weight evolution, which yields *R*^2^ = 0.937. Some deviations exist due to the slightly nonlinear switching behavior of the individual devices at high and low weights (Figures 1C,D) at the extremes of the potentiation and depression range; a recent formulation using p(gT2-TT) organic materials with a larger dynamic range could overcome this problem (Melianas et al., 2020). This nonlinearity is present in the experiment (Figure 3) and the simulation based on device data (Figure 4A), but not present in the “ideal” simulation at floating point precision (Figure 4B), making it more difficult to experimentally move the weights further away from 0.

Figures 4C,D uses the discrete-value weight updates based on the same cross-entropy cost function. While the overall trend of the simulated weight evolution (Figure 4C) is similar to that of experiment (Figure 3C), some deviations are present such that *R*^2^ = 0.876. The origin of this deviation arises from slight deviations in the weights at the threshold of accuracy may result in an extra relatively large discrete weight update. In the ideal numerical case in Figure 4D, *R*^2^ = 0.896, about equal to that in Figure 4C. Unlike the case for the continuous-value updates, there is less effect of nonlinearity because the weight range is much narrower (+/−1.5 vs. +/−4.0), placing the devices in a conductance range where they operate more linear and ideal.

The results presented in Figures 3, 4 summarize the successful demonstration of *in situ* training using an ECRAM array: hardware parallel array training accompanied by a close match between device-level experiments and crossbar simulations.

### Analysis of Experimental Training Accuracy

**FIGURE 5:**
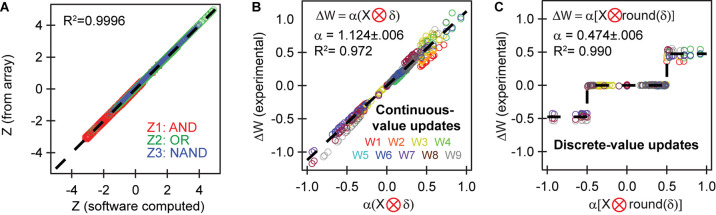
Accuracy of the analog matrix operations. The X axes computes the expected values for the vector matrix multiply and outer product update using software during post-processing. The Y axes plots the obtained value for *Z* and Δ*W* experimentally measured from the crossbar. **(A)** Vector-matrix multiply shows near-perfect agreement. **(B,C)** Outer product updates are conducted very accurately in accordance with defined cost functions and learning rules, with R^2^ over 97%, showing excellent goodness of fit. This result explains the strong convergence between the numerical and experimental results in Figure 4. The values for α were fit from the experimental vs. simulated outer product updates.

The excellent agreement between simulated and experimental training is a result of the excellent accuracy of the array to conduct the vector-matrix multiply and the outer product update. Figure 5A plots the accuracy of vector matrix multiply by comparing the **Z** obtained in analog manner directly by measuring the currents in each column of the crossbar and the **Z** obtained by measuring the weight of each device, then adding them in software using floating-point precision during post-processing. Our results show extremely high accuracy with an R^2^ value of 0.9996, suggesting that the network can conduct inference with essentially perfect accuracy.

Figures 5B,C shows the accuracy of the parallel outer product updates for the continuous and discrete-value updates by plotting the expected Δ*W* computed from the error δ, against the actual weight change computed by measuring the synaptic weight before and after the weight update. R^2^ here ranges from 0.97 for continuous-value updates and 0.99 for discrete-value updates. This ensures that any desired weight update can be realized with very high accuracy. The high accuracy of outer product updates demonstrated here is a vast improvement over past works (Fuller et al., 2019a; Kim et al., 2019) in both individual device switching behavior as well as device-to-device variation, and is essential for reaching convergence. As we show in simulation later, this quality strongly facilitates achieving rapid convergence in more complex problems involving hidden layers.

### Analysis of Nonlinearity and Variability

We quantitatively compare the performances of ECRAM devices with the metrics identified by Sun and Yu (2019). The key metrics for training are the degree of nonlinearity, which defines to what extent the weight (conductance) change Δ*W* changes with the weight *W*, the cycle-to-cycle variation, determined by the reproducibility of the switching processes within a device, and the device-to-device variation, which sets the variation in the nonlinearity between devices.

We applied the analytical methodology proposed by Sun and Yu (2019) to classify three additional classes of nonvolatile memory: the ECRAM cells used in this work, the SONOS floating-gate memory conducted in Agarwal et al. (2019), and the TaOx resistive random access memory (RRAM) cells from Bennett et al. (2019a). Table 1 summarizes these results.

**TABLE 1:** Quantitative comparison of non-volatile devices for training based on the analysis protocol developed by Sun and Yu (2019).

Device	Nonlinearity (positive/negative updates)	Cycle-to-cycle variation	Device-to-device variation in nonlinearity	Notes
TaOx/HfOx RRAM	+0.04, −0.63	3.7%	Not available	Data from Wu et al. (2018); Analysis by Sun and Yu (2019)
SiGe epiRAM	+0.5/−0.5	2%	Not available	Data from Choi et al. (2018); Analysis by Sun and Yu (2019)
HZO FeFET	+1.75/+1.46	0.5%	Not available	Data from Gonugondla et al. (2018); Analysis by Sun and Yu (2019)
ECRAM (this work)	+0.7/−0.12	0.023%	0.183/0.026	Based on 9 devices from Figure 1
SONOS (*V*_*G*_ = 2.6V)	+1.59/−2.22	0.23%	Not available	Data from Agarwal et al. (2019)
TaOx ReRAM	+668/−51.7	11.20%	216/23.0	Data from 9 most linear devices from Bennett et al. (2019a)

*The analysis from the first three rows were conducted by Sun and Yu (2019). Both the data and analysis for ECRAM are contained in this work. The data for the last two columns were previously published, but the analysis was conducted for the first time here. The two variation columns are computed at 1 standard deviation. We note that the cycle-to-cycle variation is normalized by the full conductance range. Thus, a 1% value here suggests that the average variation (1 standard deviation) equals the difference in conductance between adjacent analog states for a 100-state memory cell.*

According to Table 1, the nonlinearity of ECRAM is comparable to that of the best published TaOx/HfOx RRAM and SiGe epiRAM devices; the other devices show higher nonlinearity. ECRAMs excels in its low cycle-to-cycle variation because the electrochemical insertion and extraction of ions is much more deterministic and predictable than the stochastic formation of memristive filaments (Ielmini and Wong, 2018; Li et al., 2020a). As seen in Figure 1D, the Δ*W* values for ECRAM cells occur in a very narrow region.

Despite the excellent metrics of ECRAM with regards to nonlinearity and device-to-device variation, two additional factors need to be considered when interpreting these results. First, the ECRAM gate and channels are 750 μm × 2,000 μm each. Smaller devices, including ones scaled to sub-micron dimensions, were shown in past works (Tang et al., 2018; Melianas et al., 2020), but it has yet to be tested how downscaled devices perform relative to the metrics shown in Table 1. Second, ECRAM cells in this work have a high electronic conductance, which results in high read energies. Other organic (Fuller et al., 2019a) and inorganic (Tang et al., 2018; Li et al.,2020a,b) ECRAM cells with a channel conductance of ∼10 MΩ have been shown; however, the device-to-device variation between devices have not been studied in detail.

### Array-Level Simulations of Training

Having validated the agreement between simulated and experimental results on a small array in Figure 4, we use simulation to systematically compare the classification accuracy of ECRAM and TaOx memristors at solving the three simple logic gates with a cross-entropy cost function and continuous-valued weight updates. Our numerical simulations are a significant advance over previous work by elucidating the effect of device-to-device variation, instead of only utilizing the properties of a single device. For the logic gates, we simulate a small 3 × 3 crossbar, identical to experiment, with 100 different initial conductance values as the “seed” conditions, and plot what percentage of the “seeds” yields full accuracy on all three logic gates. Because these logic gates are easier to classify than most machine learning problems, we use this more stringent definition of “percent full accuracy.”

**FIGURE 6:**
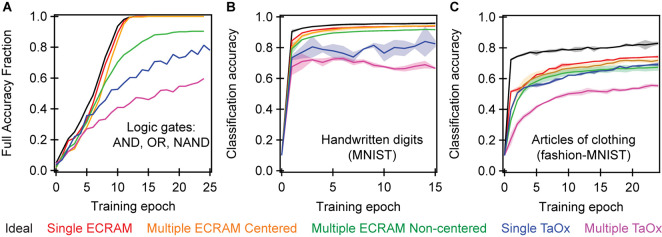
Simulated accuracy of (A) logic gates, (B) handwritten digits classification, and (C) articles of clothing classification. **(A)** used a single-layer 3 × 3 perceptron network like experiment; the full accuracy fraction denotes the fraction of seed values that are able to achieve 100% accuracy for all twelve permutations of for the three logic gates designed by Figure 2A. Two multi-layer perceptron networks, each with a single hidden layer, were simulated in **(B)** and **(C)**. The accuracy denotes the fraction of the data that was correctly classified. The handwritten digits classification **(B)** used a 65 × 36 and a 37 × 10 crossbar, while the articles of clothing classification **(C)** used a 785 × 400 and a 401 × 10 crossbar. The single ECRAM and single TaOx memristor cases use the switching properties from a single device and assumes all devices are identical in the array. The multiple ECRAM and multiple TaOx cases account for device-to-device variation using interpolation between multiple device probabilistic maps (look-up-tables), as described in Agarwal et al. (2016) and Bennett et al. (2019a). Centered simulations correct for differences in the equilibrium conductance of the device, so they are all oriented around a single conductance value (as was done experimentally with bias devices) while the non-centered simulation does not perform this correction. All simulations show significant improvement of ECRAM over TaOx, as well as the importance of accounting for device-to-device variations. The shaded regions represent two standard deviations of the simulation, each conducted over 100 random “seed” initial weights.

Figure 6A shows the results of several simulations to show the effects of non-ideal behavior within a single device as well as the variation between devices. The first “ideal” simulation is the software implementation of the algorithm at floating point precision: in this simulation, all logic gates converge to 100% accuracy. The second simulation takes the switching behavior of a single ECRAM cell, which we denote as a lookup table (e.g., device W1 in Figure 1), and replicates that behavior for all devices. This “single ECRAM” accounts only for the effects of asymmetric nonlinearity and cycle to cycle variability, and the simulated crossbar essentially achieves the numerical limit at floating-point precision. Most past numerical simulations of crossbar accuracy in ECRAM generally present these two results.

The third simulation, denoted as “multiple ECRAM, centered,” accounts for some device-to-device variability by using all nine lookup tables. This simulation accounts for variation in switching behavior, such as certain devices being more sensitive or more nonlinear than others. However, this simulation also “centers” the range of all devices around a fixed value, as was done in Figure 1C. This can be realized in one of two ways: one approach is to reduce device-to-device variability by improved ECRAM fabrication process so that all ECRAM cells would have the same equilibrium conductance; a second method is to pair each ECRAM with a calibrated offset resistor, as was done in this work (Figures 1, 2B). We propose that the second method can be realized on-chip by using a paired non-volatile memory element like a memristor, that is initially programmed to approximately equal the equilibrium conductance of the ECRAM cell and not changed afterward. This memory element also serves to enable negative weights much like the bias resistor used in this work (Figure 1A). Our results show a slightly slower convergence in this case, but the accuracy nonetheless converges consistently. This simulation is similar to the conducted experiment (Figures 3A,B) and paired simulations (Figure 4A): the 8 epochs needed for half the simulated seeds to convergence is very similar to the ∼9 epochs needed on average for the six experimental seeds to converge in Figure 3B. All simulations in this section assume continuous-valued weight updates.

The fourth simulation, denoted as “multiple ECRAM, non-centered,” removes the “centering” process and accounts for the variable range of the different ECRAM cells. As shown by the values of the bias resistors in Figure 1, the devices’ equilibrium conductance ranges from 2.1 to 2.6 mS. This simulation shows that only ∼90% of the initial seeding conditions will converge to full accuracy. Unlike experiments and the third simulation, this fourth simulation does not fully converge because it chooses a common center-point for all devices; in other words, it is equivalent to using the same bias resistor, like 2.4 mS, for all synapses. Due to the different physical conductance ranges of various devices, the switching properties for some devices around the global centerpoint are different from that of the other devices around the same value. This results in non-uniform and nonlinear weight update behavior. The reduction in accuracy here shows the crucial importance of having devices with the same center conductance, or finding a method to correct for this such as by using bias resistors.

We also conduct two simulations using TaOx memristors based on the switching properties published previously (Bennett et al., 2019a). As shown in Table 1, this dataset contains both nonlinearity and device-to-device variation data. This result shows that ∼ 80% of the initial conditions fully converge when replicating the properties of a single device (“single TaOx”) and ∼50% fully converge when accounting for device-to-device variation (“many TaOx”). This simulation shows that ECRAM has significant advantages in terms of training accuracy even in the case of simple logic gate functions. It further shows that attaining full accuracy for the 100 simulated seeds in logic gates, as done experimentally with ECRAM, is a nontrivial task that requires excellent individual device switching properties.

We simulate the accuracy of the same devices for image classification of handwritten digits, using a downsampled version of the MNIST database known as the UCI Optical Character Recognition (OCR) task (Alpaydin and Kaynak, 1998), and articles of clothing, from the more recently developed Fashion-MNIST database (Xiao et al., 2017) in Figures 6B,C. Each task contains ten categories or classes. Because these images are harder to classify than the logic gate inputs, we plot the average fraction of correctly identified solutions for the 100 simulated seeds, as opposed to the fraction of simulated seeds that achieve 100% accuracy. The chosen two-layer network topology consists of a 65 × 36 crossbar followed by a 37 × 10 crossbar – the outputs from the first crossbar, plus a bias input, are used as inputs for the second crossbar. A 95% accuracy is achieved on the handwritten digits task in the numeric limit. All ECRAM simulations yield above 91% accuracy, while the TaOx simulations yield significantly lower accuracies. The fashion-MNIST network yields just 83% accuracy at the numerical limit, due to the greater complexity and nonlinearity of the classification problem, with progressively lower accuracies for ECRAM and TaOx. This simulation also utilized a two layer network, but this time consisting of a 785 × 400 and a 401 × 10 crossbar. Once again, the outputs form the first crossbar plus a bias input are used as inputs for the second crossbar.

All simulations show that ECRAM outperforms TaOx under any configuration. However, TaOx simulations appear more inaccurate in handwritten digits than in articles of clothing because the handwritten digits require higher learning rates, such that the nonlinear behavior of the filamentary memristors more significantly decrease the accuracy (see methods for exact numbers). In contrast, articles of clothing utilize lower learning rates, and a lower theoretical accuracy using fully connected networks. The lower learning rates are more resilient to device nonlinearity, such that the single-device TaOx accuracy nears that of ECRAM. This work further work shows the importance of multiple metrics in characterizing the accuracy of different devices.

Our results further show that device-to-device variation plays a significant role in the accuracy of in-situ training networks. In memristors, device-to-device variation is an intrinsic property relating to the discrete, stochastic nature of atomic point defects within the conductive filament (Yu et al., 2013; Ielmini and Wong, 2018). For ECRAM, our observed device-to-device variation likely result from an unoptimized fabrication process. Specifically, the ECRAMs in this work were fabricated using solution-processing followed by lift-off (Khodagholy et al., 2011), which enables rapid prototyping but is not well suited for reduced device-to-device variability. This device-to-device variability will likely be more significant for devices with lower conductances, such as the nano-Siemen devices shown in past work (Fuller et al., 2019a), which would likely result in lower weight tuning and training accuracy. The lift-off method can be replaced by more advanced lithography techniques to improve device yield and areal density.

Our simulations again highlight two different types of device-to-device variation within ECRAM, one primarily behavioral and relating to differences in slightly distinct switching properties among each device’s probabilistic switching map (from “single ECRAM” to “many ECRAM, centered”), and the other relating to range-derived differences in the equilibrium conductance (from “many ECRAM, centered” to “many ECRAM, non-centered”). While both should improve with better fabrication and processing, we anticipate the latter equilibrium conductance is simpler to tackle because the conductance is determined by PEDOT:PSS film uniformity across the wafer die. In contrast, the switching variability depends on the uniformity in the entire multilayer PEDOT:PSS/electrolyte stack and the associated charge transfer kinetics within this stack, which may be more difficult to control. If ECRAM provides the elusive memory element for parallel *in situ* learning, our approach paves the way toward massively parallel training of artificial neural networks at unprecedented levels of energy efficiency.

### ECRAM for Deep and Spiking Neuronal Learning Methods

We further consider how the *in situ* learning approaches we present in this work could be extended to spiking neural networks (SNNs). Unlike multilayer perceptron networks (e.g., deep neural networks and convolutional neural networks) that transmit information at each propagation cycle, neurons in SNNs are only activated when their activation reaches the threshold potential. By encoding information more sparsely in the temporal domain and restricting the analog requirements for information transmission, SNNs are widely considered more likely to achieve the energy efficiency and error tolerance of biological computing system (Wang et al., 2020). Spike time-dependent plasticity (STDP) like functionality have been demonstrated in organic and inorganic ECRAM (van de Burgt et al., 2017; Sharbati et al., 2018; Li et al., 2020b). While STDP or STPD-like rules do not directly implement stochastic gradient descent backpropagation, they effectively sample an input and may approximate a statistical method known as expectation maximization (Nessler et al., 2013).

Numerical approaches to derive loss functions based on spike learning have been proposed (Shrestha et al., 2019), although it is not clear how well these approaches scale with multiple spiking hidden layers. However, the complexity of implementing these rules is typically simpler in nanosynapses, since STDP does not require synaptic linearity, and may even be able to exploit some degree of synaptic non-linearity (Querlioz et al., 2013). Yet STDP-style learning systems still require minimum synaptic analog resolution, as well as symmetry between programming/update modes. Shallow or sampling networks can effectively implement STDP learning with 4–5 bit resolution (Pfeil et al., 2012; Woods et al., 2015). In addition, the requirements may be relaxed even further when using a technique known as the Linear Solutions of Higher Dimensional Interlayers to improve the linear separability of a machine learning (Tapson et al., 2013), which was recently demonstrated for emerging device crossbar based systems (Bennett et al., 2019b).

Although the stringency requirements for implementing SNN learning may be lower than for stochastic gradient descent, this study’s focus on reliable analog switching, including realistic considerations of device-to-device and cycle-to-cycle variation, will be an asset to future works on this topic. For instance, our analysis that synaptic conductance range centering is critical to implementing effective parallel updates may be equally advantageous to realizing SNN layer-by-layer learning that is parallelizable. A lower bound estimate of cycle-to-cycle noise tolerable on STDP learning has not been established in the literature, but may be better interrogated given our approach. Conversely, from the perspective of device optimization for efficient SNN implementation, the analog depth, low cycle-to-cycle noise, and good mode symmetry of ECRAM devices suggest these devices will be strong candidates for hardware SNNs. In contrast, while phase-change memory has recently been used to physically realize STDP learning they have substantial asymmetry between SET and RESET modes (Ambrogio et al., 2016). This creates additional circuit overhead for accessing synapses on dense crossbars and damps the maximal efficiency possible with local learning rules. STDP, short term potentiation, and paired pulse facilitation has already been demonstrated in organic ECRAM devices similar to those presented here (van de Burgt et al., 2017). Importantly, ion transport kinetics and material composition play decisive roles in determining the temporal response and, therefore, are likely to similarly affect efficiency and accuracy of the spiking timing based learning algorithms. Our work therefore strongly encourages further investigation of three-terminal ECRAM devices for modern SNN learning.

## Conclusion

In summary, we experimentally achieve parallel *in situ* training using organic ECRAM synapses with high accuracy, a necessary step toward realizing efficient and accurate hardware training accelerators. Moreover, we show unprecedented reproducibility between the simulated and experimental training results, not just in the number of epochs to convergence but the exact evolution of the weight of each synapse. By experimentally demonstrating training using outer product updates that are consistent with numerical simulations, our work implies the potential of ECRAM cells to ultimately contribute to high accuracy in neural network training accelerators, and affirms the ability of our software methodology to contribute to leading toward this important goal.

## Materials and Methods

### Fabrication of ECRAM Cells

The ECRAM devices were patterned as reported previously (Fuller et al., 2019a; Melianas et al., 2020). Briefly, Ti(8 nm)/Au(50 nm) electrodes were patterned on Si wafers with 1 μm thick SiO_2_ using e-beam evaporation. The wafers were then coated with 1.5 μm parylene C as the insulating layer which was crosslinked with the adhesion promoter 3-(trimethoxysilyl)propyl methacrylate. After coating the first parylene C layer, a dilute soap solution (3% Micro-90 in H_2_O) was spincoated on top, followed by coating another 1.5 μm layer of parylene C. The wafers were then coated with 75 nm Ti and were subsequently patterned and dry-etched to define the channel, gate, and electrode pad areas. In this work, each wafer die had 8 devices. Before PEDOT:PSS deposition, the wafer dies were cleaned using 5 min sonication in isopropanol followed by 5 min UV-Ozone treatment. PEDOT:PSS was spincoated on the wafer dies in ambient at 1000 rpm for 2 min and baked at 120°C for 20 min. The top parylene C layer was then peeled off, leaving PEDOT:PSS only in the photolithographically defined channel, gate, and contact pad regions. Before electrolyte deposition, the wafer die was rinsed in H_2_O to remove residual Micro-90. The ion gel electrolyte (Melianas et al., 2020) was then drop-cast on top of each device using a micropipette.

Finally, the chips were wire-bonded onto a PLCC-68 chip carrier and breakout board to address the many circuit leads of the board.

### Circuit Design

The circuit was implemented on a four-layer printed circuit board (PCB) fabricated by Gorilla Circuits (San Jose, CA, United States). Molex connectors were used to connect the PCB to the wire-bonded ECRAM chips. All other active (e.g., CMOS switches, op-amps) and passive (e.g., discrete resistors, capacitors) circuit components are also placed and wired using the printed circuit board. In addition to the circuit and components shown in Figure 2B, the PCB also includes a current subtraction circuit (Figure 1B) and an operational transconductance amplifier for each output column in order to convert the multiply-and-accumulate output current into a voltage that can be logged by a data acquisition instrument (NI-DAQ).

The three analog inputs X_1__–__3_ were connected to three analog outputs from a NI-DAQ, PCIe-6363; the read voltage is typically ∼50 mV. The write voltage (*V*_*W*_ = +/−1V), common to all cells, were also connected to a fourth analog output from the NI-DAQ. The output voltage from the operational transconductance amplifier were connected to the analog inputs of a NI-DAQ. The NI-DAQ was controlled using a custom-built LabVIEW software.

### Crossbar Array Control

A custom-built Python package served as the user interface with the LabVIEW software used to control the DAQ. Each time step is 0.1 s. To conduct inference, a 50-mV read voltage was applied to each row *i* where *X*_*i*_ = 1, and 0-mV applied when *X_*i*_* = 0 for 10 ms. The currents read at the bottom represents the results of the multiply-and-accumulate function. Digital outputs D_1__–__6_ were held at low (0V) to keep the switches OFF and prevent leakage current. To conduct array training, the voltage V_W_ was held at either +1 for depression or −1 for potentiation. The outer production selection is conducted through the pulse widths of the digital switches. D_1__–__3_ has a pulse width of 10 ms when *X_1__–__3_* = 1, and 0 ms when *X_1__–__3_* = 0. The pulse width of D_4__–__6_ is proportional to α⋅δ for continuous-value updates and α⋅*round*(δ) for discrete-value updates. A training epoch is defined as iterating this process through all four training examples.

Finally, to measure the conductance of individual array elements, 10-ms read voltage pulses were separately applied to each row of the crossbar. Because this voltage is applied to only one of X1, X2, or X3 at a time, the read currents at the bottom equals $I_{i}-I_{i}^{b\,i\,a\,s}$, where *I_i* is the current through ECRAM cell *i* while $I_{i}^{b\,i\,a\,s}$ is the current through the paired bias resistor. We note that this step is a diagnostic measure, and the results were only recorded for post-processing and data analysis, and not using during *in situ* training.

### Crossbar Simulations

Crossbar simulations were conducted on an expanded version of the open-source Sandia CrossSim simulation package, which is written in Python and allows for physics-realistic simulation of neural network accelerators. For the *in situ* learning case, which is the focus of this work, CrossSim instantiates neural cores with a variety of parameter corresponding to both device properties (conductance evolution behavior) as well as general neural network properties, such as network topology, choice of task, and learning rates. In order to effectively match the simulations with experiment, a custom set of look-up-tables (LUT) have been constructed by using experimentally derived sets of repeated ramped pulses. The LUT for ECRAM are given in Figure 1D, while the LUT for TaOx were taken from past work (Bennett et al., 2019a). This probabilistic matrix of look-up-tables is represented here as ΔG_i_(G_i_), signifying that the change in conductance is a function of the present conductance. Then, when conducting backpropagation, globally requested updates applied to the logical cores via an outer-product-update (OPU) are individualized to simulated devices in the crossbar. The change in simulated weight is proportional to the product of ΔG_i_(G_i_) and the results of the OPU [**X**⊗δ or **X**⊗*round*(δ)].

The simulations in Figures 4A,C were conducted by initializing, or seeding, the simulations at the same weights as the experiment, and using the LUT for each device. The ideal numerical simulations in Figures 4B,D were conducted assuming perfectly linear and symmetric devices: all ΔG_i_ are identical for all device and all G.

The simulations in Figure 6 used 100 random initial seed values. Figure 6A plot the fraction of seed values that will achieve 100% classification accuracy of the twelve permutations of the logic gates trained in experiment (Figure 2A). Figures 6B,C plots average classification accuracy of the data set across all seeds. The learning rate for handwritten digits were 0.01 for numeric, 0.006 for single lookup table, and 0.012 for multiple lookup tables. The learning rate for articles of clothing were 0.001 for numeric, 0.0001 for single lookup table, and 0.00015 for multiple lookup tables.

The “ideal numerical” simulations in Figure 6 assumes that ΔG_i_ is equal for all devices and conductance states. The single-ECRAM and single-TaOx uses the same LUT from one device to simulate all device properties in the array. The multiple-ECRAM for logic gates uses the same nine LUT from the experiment (Figure 1D). The other multiple-ECRAM and all multiple TaOx simulations picked random LUT from the dataset, with each simulated synapse drawing a random LUT. More details on CrossSim’s methodology and details of parameterized neural core operations can be found at: https://cross-sim.sandia.gov/_assets/documents/crosssim_manual.pdf.

## Data Availability Statement

The datasets presented in this study can be found in online repositories. The names of the repository/repositories and accession number(s) can be found below: Materials Commons 2.0: https://doi.org/10.13011/m3-13da-my32.

## Author Contributions

YL, EF, and AT conceived the project. AM fabricated the ECRAM devices. HT, EI, and YL designed the printed circuit board. EI, YL, and EF wrote the firmware and software for the measurements. YL conducted the measurements and analyzed the results. TX and CB conducted the simulations. All authors contributed to the writing of the manuscript.

## Conflict of Interest

YL, TX, CB, EI, HT, MM, EF, and AT were employed at Sandia National Laboratories by NTESS. The authors declare that this study received funding from the sources listed in the “funding sources” section. Sandia National Laboratories/NTESS verified that this study contains no classified information in order to approve its submission for publication. Otherwise, the funders were not involved in the study design, collection, analysis, interpretation of data, the writing of this article, or the decision to submit it for publication. The handling editor declared a past co-authorship with several of the authors AM, AT, AS, and EF.

## References

[B1] Agarwal S. Garland D. Niroula J. Jacobs-Gedrim R. B. Hsia A. Van Heukelom M. S. (2019). Using floating-gate memory to train ideal accuracy neural networks. *IEEE J. Explor. Solid State Comput. Devices Circuits* 5 52–57. 10.1109/JXCDC.2019.2902409

[B2] Agarwal S. Plimpton S. J. Hughart D. R. Hsia A. H. Richter I. Cox J. A. (2016). “Resistive memory device requirements for a neural algorithm accelerator,” in *Proceedings of the 2016 International Joint Conference on Neural Networks (IJCNN)*, Vancouver, BC, 929–938. 10.1109/IJCNN.2016.7727298

[B3] Alibart F. Zamanidoost E. Strukov D. B. (2013). Pattern classification by memristive crossbar circuits using ex situ and in situ training. *Nat. Commun.* 4:2072. 10.1038/ncomms3072 23797631

[B4] Alpaydin E. Kaynak C. (1998). *Optical Recognition of Handwritten Digits Data Set.* UCI Machine Learning Repository. Oakland, CA: University of California.

[B5] Ambrogio S. Ciocchini N. Laudato M. Milo V. Pirovano A. Fantini P. (2016). Unsupervised learning by spike timing dependent plasticity in phase change memory (PCM) synapses. *Front. Neurosci.* 10:56. 10.3389/fnins.2016.00056 27013934 PMC4781832

[B6] Ambrogio S. Narayanan P. Tsai H. Shelby R. M. Boybat I. Di Nolfo C. (2018). Equivalent-accuracy accelerated neural-network training using analogue memory. *Nature* 558 60–67. 10.1038/s41586-018-0180-5 29875487

[B7] Bayat F. M. Prezioso M. Chakrabarti B. Nili H. Kataeva I. Strukov D. (2018). Implementation of multilayer perceptron network with highly uniform passive memristive crossbar circuits. *Nat. Commun.* 9:2331. 10.1038/s41467-018-04482-4 29899421 PMC5998062

[B8] Bennett C. H. Garland D. Jacobs-Gedrim R. B. Agarwal S. Marinella M. J. (2019a). “Wafer-Scale TaOx device variability and implications for neuromorphic computing applications,” in *Proceedings of the 2019 IEEE International Reliability Physics Symposium*, (Piscataway, NJ: IEEE). 10.1109/IRPS.2019.8720596

[B9] Bennett C. H. Parmar V. Calvet L. E. Klein J. O. Suri M. Marinella M. J. (2019b). Contrasting advantages of learning with random weights and backpropagation in non-volatile memory neural networks. *IEEE Access* 7 73938–73953. 10.1109/ACCESS.2019.2920076

[B10] Burr G. W. Shelby R. M. Sebastian A. Kim S. Kim S. Sidler S. (2017). Neuromorphic computing using non-volatile memory. *Adv. Phys. X* 2 89–124. 10.1080/23746149.2016.1259585

[B11] Cai F. Correll J. M. Lee S. H. Lim Y. Bothra V. Zhang Z. (2019). A fully integrated reprogrammable memristor– CMOS system for efficient multiply–accumulate operations. *Nat. Electron.* 2 290–299. 10.1038/s41928-019-0270-x

[B12] Choi S. Tan S. H. Li Z. Kim Y. Choi C. Chen P. Y. (2018). SiGe epitaxial memory for neuromorphic computing with reproducible high performance based on engineered dislocations. *Nat. Mater.* 17 335–340. 10.1038/s41563-017-0001-5 29358642

[B13] Emelyanov A. V. Lapkin D. A. Demin V. A. Erokhin V. V. Battistoni S. Baldi G. (2016). First steps towards the realization of a double layer perceptron based on organic memristive devices. *AIP Adv.* 6:111301. 10.1063/1.4966257

[B14] Fuller E. J. El Gabaly F. Léonard F. Agarwal S. Plimpton S. J. Jacobs-Gedrim R. B. (2017). Li-Ion synaptic transistor for low power analog computing. *Adv. Mater.* 29:1604310. 10.1002/adma.201604310 27874238

[B15] Fuller E. J. Keene S. T. Melianas A. Wang Z. Agarwal S. Li Y. (2019a). Parallel programming of an ionic floating-gate memory array for scalable neuromorphic computing. *Science* 364 570–574. 10.1126/science.aaw5581 31023890

[B16] Fuller E. J. Li Y. Bennet C. Keene S. T. Melianas A. Agrawal S. (2019b). Redox transistors for neuromorphic computing. *IBM J. Res. Dev.* 63:9:1–9:9. 10.1147/JRD.2019.2942285

[B17] Gokmen T. Vlasov Y. (2016). Acceleration of deep neural network training with resistive cross-point devices: design considerations. *Front. Neurosci.* 10:333. 10.3389/fnins.2016.00333 27493624 PMC4954855

[B18] Gonugondla S. K. Kang M. Shanbhag N. (2018). “A 42pJ/decision 3.12TOPS/W robust in-memory machine learning classifier with on-chip training,” in *Proceedings of the 2018 IEEE International Solid-State Circuits Conference*, San Francisco, CA, 490–492. 10.1109/ISSCC.2018.8310398

[B19] Guo X. Bayat F. M. Bavandpour M. Klachko M. Mahmoodi M. R. Prezioso M. (2017). “Fast, energy-efficient, robust, and reproducible mixed-signal neuromorphic classifier based on embedded NOR flash memory technology,” in *Proceedings of the 2017 International Electron Devices Meeting*, San Francisco, CA. 17:151–17:154. 10.1109/IEDM.2017.8268341

[B20] Hu M. Graves C. E. Li C. Li Y. Ge N. Montgomery E. (2018). Memristor-Based analog computation and neural network classification with a dot product engine. *Adv. Mater.* 30:1705914. 10.1002/adma.201705914 29318659

[B21] Ielmini D. Wong H. S. P. (2018). In-memory computing with resistive switching devices. *Nat. Electron.* 1 333–343. 10.1038/s41928-018-0092-2

[B22] Jacobs-Gedrim R. B. Agarwal S. Knisely K. E. Stevens J. E. van Heukelom M. S. Hughart D. R. (2017). “Impact of linearity and write noise of analog resistive memory devices in a neural algorithm accelerator,” in *Proceedings of the 2017 IEEE International Conference on Rebooting Computing*, (Piscataway, NJ: IEEE), 17413472. 10.1109/ICRC.2017.8123657

[B23] Keene S. T. Melianas A. Fuller E. J. Van De Burgt Y. Talin A. A. Salleo A. (2018a). Optimized pulsed write schemes improve linearity and write speed for low-power organic neuromorphic devices. *J. Phys. D Appl. Phys.* 51:224002.

[B24] Keene S. T. Melianas A. Van de Burgt Y. Salleo A. (2018b). Mechanisms for enhanced state retention and stability in redox-gated organic neuromorphic devices. *Adv. Electron. Mater.* 5:1800686. 10.1002/aelm.201800686

[B25] Khodagholy D. Gurfinkel M. Stavrinidou E. Leleux P. Herve T. Sanaur S. (2011). High speed and high density organic electrochemical transistor arrays. *Appl. Phys. Lett.* 99 99–102. 10.1063/1.3652912

[B26] Kim S. Todorov T. Onen M. Gokmen T. Bishop D. Solomon P. (2019). “Metal-oxide based, CMOS-compatible ECRAM for deep learning accelerator,” in *Proceedings of the 2019 International Electron Devices Meeting*, San Francisco, CA, 847–850. 10.1109/IEDM19573.2019.8993463

[B27] Lecun Y. Bengio Y. Hinton G. (2015). Deep learning. *Nature* 521 436–444. 10.1038/nature14539 26017442

[B28] Lee S.-T. Lee J.-H. (2020). Neuromorphic computing using NAND flash memory architecture with pulse width modulation scheme. *Front. Neurosci.* 14:571292. 10.3389/fnins.2020.571292 33071744 PMC7530297

[B29] Li C. Belkin D. Li Y. Yan P. Hu M. Ge N. (2018a). Efficient and self-adaptive in-situ learning in multilayer memristor neural networks. *Nat. Commun.* 9:2385. 10.1038/s41467-018-04484-2 29921923 PMC6008303

[B30] Li C. Hu M. Li Y. Jiang H. Ge N. Montgomery E. (2018b). Analogue signal and image processing with large memristor crossbars. *Nat. Electron.* 1 52–59.

[B31] Li Y. Fuller E. J. Asapu S. Agarwal S. Kurita T. Yang J. J. (2019). Low-Voltage, CMOS-Free synaptic memory based on Li_X_TiO_2_ redox transistors. *ACS Appl. Mater. Interfaces* 11 38982–38992. 10.1021/acsami.9b14338 31559816

[B32] Li Y. Fuller E. J. Yoo S. Ashby D. S. Bennett C. H. Horton R. D. (2020a). Filament-free bulk resistive memory enables deterministic analogue switching. *Adv. Mater.* 32:2003984. 10.1002/adma.202003984 32964602

[B33] Li Y. Lu J. Shang D. Liu Q. Wu S. Wu Z. (2020b). Oxide−Based electrolyte−gated transistors for spatiotemporal information processing. *Adv. Mater.* 32:2003018. 10.1002/adma.202003018 33079425

[B34] Lin Y. P. Bennett C. H. Cabaret T. Vodenicarevic D. Chabi D. Querlioz D. (2016). Physical realization of a supervised learning system built with organic memristive synapses. *Sci. Rep.* 6:31932. 10.1038/srep31932 27601088 PMC5013285

[B35] Marinella M. J. Member S. Agarwal S. Hsia A. Jacobs-gedrim R. Niroula J. (2017). Multiscale co-design analysis of energy, latency, area, and accuracy of a ReRAM analog neural training accelerator. *IEEE J. Emerg. Sel. Top. Circuits Syst.* 8 86–101. 10.1109/JETCAS.2018.2796379

[B36] Melianas A. Quill T. J. LeCroy G. Tuchman Y. Loo H. v. Keene S. T. (2020). Temperature-resilient solid-state organic artificial synapses for neuromorphic computing. *Sci. Adv.* 6:eabb2958. 10.1126/sciadv.abb2958 32937458 PMC7458436

[B37] Nandakumar S. R. Le Gallo M. Piveteau C. Joshi V. Mariani G. Boybat I. (2020). Mixed-Precision deep learning based on computational memory. *Front. Neurosci.* 14:406. 10.3389/fnins.2020.00406 32477047 PMC7235420

[B38] Nawrocki R. A. Voyles R. M. Shaheen S. E. (2016). A mini review of neuromorphic architectures and implementations. *IEEE Trans. Electron Devices* 63 3819–3829. 10.1109/TED.2016.2598413

[B39] Nessler B. Pfeiffer M. Buesing L. Maass W. (2013). Bayesian computation emerges in generic cortical microcircuits through spike-timing-dependent plasticity. *PLoS Comput. Biol.* 9:1003037. 10.1371/journal.pcbi.1003037 23633941 PMC3636028

[B40] Park Y. Lee J. S. (2017). Artificial synapses with short- and long-term memory for spiking neural networks based on renewable materials. *ACS Nano* 11 8962–8969. 10.1021/acsnano.7b03347 28837313

[B41] Pfeil T. Potjans T. C. Schrader S. Potjans W. Schemmel J. Diesmann M. (2012). Is a 4-Bit synaptic weight resolution enough? – constraints on enabling spike-timing dependent plasticity in neuromorphic hardware. *Front. Neurosci.* 6:90. 10.3389/fnins.2012.00090 22822388 PMC3398398

[B42] Prezioso M. Hoskins B. D. Adam G. C. Likharev K. K. Strukov D. B. (2015). Training and operation of an integrated neuromorphic network based on metal-oxide memristors. *Nature* 521 61–64. 10.1038/nature14441 25951284

[B43] Querlioz D. Bichler O. Dollfus P. Gamrat C. (2013). Immunity to device variations in a spiking neural network with memristive nanodevices. *IEEE Trans. Nanotechnol.* 12 288–295. 10.1109/TNANO.2013.2250995

[B44] Sebastian A. Le Gallo M. Khaddam-Aljameh R. Eleftheriou E. (2020). Memory devices and applications for in-memory computing. *Nat. Nanotechnol.* 15 529–544. 10.1038/s41565-020-0655-z 32231270

[B45] Sharbati M. T. Du Y. Torres J. Ardolino N. D. Yun M. Xiong F. (2018). Low-Power, electrochemically tunable graphene synapses for neuromorphic computing. *Adv. Mater.* 30:1802353. 10.1002/adma.201802353 30033599

[B46] Shrestha A. Fang H. Wu Q. Qiu Q. (2019). “Approximating back-propagation for a biologically plausible local learning rule in spiking neural networks,” in *Proceedings of the ICONS ‘19th International Confernece on Neuromorphic Systems*, (New York, NY: ACM), 10. 10.1145/3354265.3354275

[B47] Sun X. Yu S. (2019). Impact of non-ideal characteristics of resistive synaptic devices on implementing convolutional neural networks. *IEEE J. Emerg. Sel. Top. Circuits Syst.* 9 570–579. 10.1109/JETCAS.2019.2933148

[B48] Sze V. Chen Y. H. Yang T. J. Emer J. S. (2017). Efficient processing of deep neural networks: a tutorial and survey. *Proc. IEEE* 105 2295–2329. 10.1109/JPROC.2017.2761740

[B49] Tang J. Bishop D. Kim S. Copel M. Gokmen T. Todorov T. (2018). “ECRAM as scalable synaptic cell for high-speed, low-power neuromorphic computing,” in *Proceedings of the 2018 International Electron Devices Meeting*, San Francisco, CA, 18:292–18:295. 10.1109/IEDM.2018.8614551

[B50] Tapson J. C. Cohen G. K. Afshar S. Stiefel K. M. Buskila Y. Wang R. M. (2013). Synthesis of neural networks for spatio-temporal spike pattern recognition and processing. *Front. Neurosci.* 7:153. 10.3389/fnins.2013.00153 24009550 PMC3757528

[B51] Tuchman Y. Mangoma T. N. Gkoupidenis P. Van De Burgt Y. John R. A. Mathews N. (2020). Organic neuromorphic devices: past, present, and future challenges. *MRS Bull.* 45 619–630. 10.1557/mrs.2020.196

[B52] van de Burgt Y. Lubberman E. Fuller E. J. Keene S. T. Faria G. C. Agarwal S. (2017). A non-volatile organic electrochemical device as a low-voltage artificial synapse for neuromorphic computing. *Nat. Mater.* 16 414–418. 10.1038/nmat4856 28218920

[B53] Wang W. Song W. Yao P. Li Y. Van Nostrand J. Qiu Q. (2020). Integration and co-design of memristive devices and algorithms for artificial intelligence. *iScience* 23:101809. 10.1016/j.isci.2020.101809 33305176 PMC7718163

[B54] Woods W. Bürger J. Teuscher C. (2015). Synaptic weight states in a locally competitive algorithm for neuromorphic memristive hardware. *IEEE Trans. Nanotechnol.* 14 945–953. 10.1109/TNANO.2015.2449835

[B55] Wu W. Wu H. Gao B. Yao P. Zhang X. Peng X. (2018). “A methodology to improve linearity of analog RRAM for neuromorphic computing,” in *Proceedings of the 2018 IEEE Symposium on VLSI Technology*, Honolulu, HI, 103–104. 10.1109/VLSIT.2018.8510690

[B56] Xia Q. Yang J. J. (2019). Memristive crossbar arrays for brain-inspired computing. *Nat. Mater.* 18 309–323. 10.1038/s41563-019-0291-x 30894760

[B57] Xiao H. Rasul K. Vollgraf R. (2017). Fashion-MNIST: a novel image dataset for benchmarking machine learning algorithms. *arXiv* [preprint] arXiv:1708.07747

[B58] Yao P. Wu H. Gao B. Eryilmaz S. B. Huang X. Zhang W. (2017). Face classification using electronic synapses. *Nat. Commun.* 8:15199. 10.1038/ncomms15199 28497781 PMC5437298

[B59] Yao P. Wu H. Gao B. Tang J. Zhang Q. Zhang W. (2020). Fully hardware-implemented memristor convolutional neural network. *Nature* 577 641–646. 10.1038/s41586-020-1942-4 31996818

[B60] Yao X. Klyukin K. Lu W. Onen M. Ryu S. Kim D. (2020). Protonic solid-state electrochemical synapse for physical neural networks. *Nat. Commun.* 11:3134. 10.1038/s41467-020-16866-6 32561717 PMC7371700

[B61] Yeon H. Lin P. Choi C. Tan S. H. Park Y. Lee D. (2020). Alloying conducting channels for reliable neuromorphic computing. *Nat. Nanotechnol.* 15 574–579. 10.1038/s41565-020-0694-5 32514010

[B62] Yu S. Gao B. Fang Z. Yu H. Kang J. Wong H. P. (2013). Stochastic learning in oxide binary synaptic device for neuromorphic computing. *Front. Neurosci.* 7:186. 10.3389/fnins.2013.00186 24198752 PMC3813892

